# Technical note: Minimizing CIED artifacts on a 0.35 T MRI‐Linac using deep learning

**DOI:** 10.1002/acm2.14304

**Published:** 2024-02-18

**Authors:** Austen N. Curcuru, Deshan Yang, Hongyu An, Phillip S. Cuculich, Clifford G. Robinson, H. Michael Gach

**Affiliations:** ^1^ Department of Radiation Oncology Washington University in St. Louis St. Louis Missouri USA; ^2^ Department of Radiation Oncology Duke University Durham North Carolina USA; ^3^ Departments of Radiology Biomedical Engineering and Neurology Washington University in St. Louis St. Louis Missouri USA; ^4^ Departments of Cardiovascular Medicine and Radiation Oncology Washington University in St. Louis St. Louis Missouri USA; ^5^ Departments of Radiation Oncology Radiology and Biomedical Engineering Washington University in St. Louis St. Louis Missouri USA

**Keywords:** artifact, deep learning, ICD, MRI

## Abstract

**Background:**

Artifacts from implantable cardioverter defibrillators (ICDs) are a challenge to magnetic resonance imaging (MRI)‐guided radiotherapy (MRgRT).

**Purpose:**

This study tested an unsupervised generative adversarial network to mitigate ICD artifacts in balanced steady‐state free precession (bSSFP) cine MRIs and improve image quality and tracking performance for MRgRT.

**Methods:**

Fourteen healthy volunteers (Group A) were scanned on a 0.35 T MRI‐Linac with and without an MR conditional ICD taped to their left pectoral to simulate an implanted ICD. bSSFP MRI data from 12 of the volunteers were used to train a CycleGAN model to reduce ICD artifacts. The data from the remaining two volunteers were used for testing. In addition, the dataset was reorganized three times using a Leave‐One‐Out scheme. Tracking metrics [Dice similarity coefficient (DSC), target registration error (TRE), and 95 percentile Hausdorff distance (95% HD)] were evaluated for whole‐heart contours. Image quality metrics [normalized root mean square error (nRMSE), peak signal‐to‐noise ratio (PSNR), and multiscale structural similarity (MS‐SSIM) scores] were evaluated. The technique was also tested qualitatively on three additional ICD datasets (Group B) including a patient with an implanted ICD.

**Results:**

For the whole‐heart contour with CycleGAN reconstruction: 1) Mean DSC rose from 0.910 to 0.935; 2) Mean TRE dropped from 4.488 to 2.877 mm; and 3) Mean 95% HD dropped from 10.236 to 7.700 mm. For the whole‐body slice with CycleGAN reconstruction: 1) Mean nRMSE dropped from 0.644 to 0.420; 2) Mean MS‐SSIM rose from 0.779 to 0.819; and 3) Mean PSNR rose from 18.744 to 22.368. The three Group B datasets evaluated qualitatively displayed a reduction in ICD artifacts in the heart.

**Conclusion:**

CycleGAN‐generated reconstructions significantly improved both tracking and image quality metrics when used to mitigate artifacts from ICDs.

## 1 INTRODUCTION

Noninvasive stereotactic arrhythmia radiotherapy (STAR) is a new treatment for patients with refractory ventricular tachycardia (VT).^1^ STAR is typically performed using cone‐beam CT image guided radiotherapy (CBCT‐IGRT) to deliver a dose of up to 35 Gy in a single fraction to the myocardial lesion that produces the arrhythmia. Currently, the treatment target needs to be expanded by 7−10 mm to account for motion, gating uncertainties, and patient set up. These margin expansions result in healthy tissues near the lesion receiving therapeutic dose to ensure complete target coverage. Larger setup margins can be avoided by keeping the tracking errors less than the intended setup margin.

Target margins and radiotoxicities could potentially be reduced using magnetic resonance imaging (MRI)‐guided radiotherapy (MRgRT).^2^ MRgRT provides real‐time target tracking and superior soft tissue contrast compared with conventional x‐ray guided Linacs. With MRgRT, the treatment beam can be gated to specific cardiac and respiratory phases without relying on surrogate signals. Balanced steady‐state free procession (bSSFP) cine sequences are currently used for real‐time MRgRT treatment but are prone to severe imaging artifacts from implantable cardioverter defibrillators (ICDs) commonly implanted in VT patients.

Several machine learning (ML) techniques were developed for image‐to‐image translation and artifact reduction. Generative adversarial networks (GANs) were successful for MRI reconstruction, denoising, super‐resolution, segmentation, motion artifact reduction, and image modality translation.^3^, ^4^, ^5^, ^6^, ^7^, ^8^, ^9^, ^10^, ^11^ However, an unsupervised learning approach is often required due to the difficulty in acquiring accurately paired image data or generating physiologically meaningful synthetic datasets. Cycle‐consistent generative adversarial network, or CycleGAN, is a widely used unsupervised deep learning architecture for image‐to‐image translation problems.^12^ CycleGAN was reported to be successful in several clinically relevant domains including CT metal artifact reduction, visual enhancement of CBCT images, and synthetic CT generation.^13^, ^14^, ^15^, ^16^ Additionally, the CycleGAN can be optimized to work for near real‐time applications.^17^

In this study, we apply and evaluate the CycleGAN to reduce ICD artifacts in bSSFP MRI cines for use in STAR.

## 2 METHODS

### 2.1 Deep learning (DL) architecture

**FIGURE 1:**
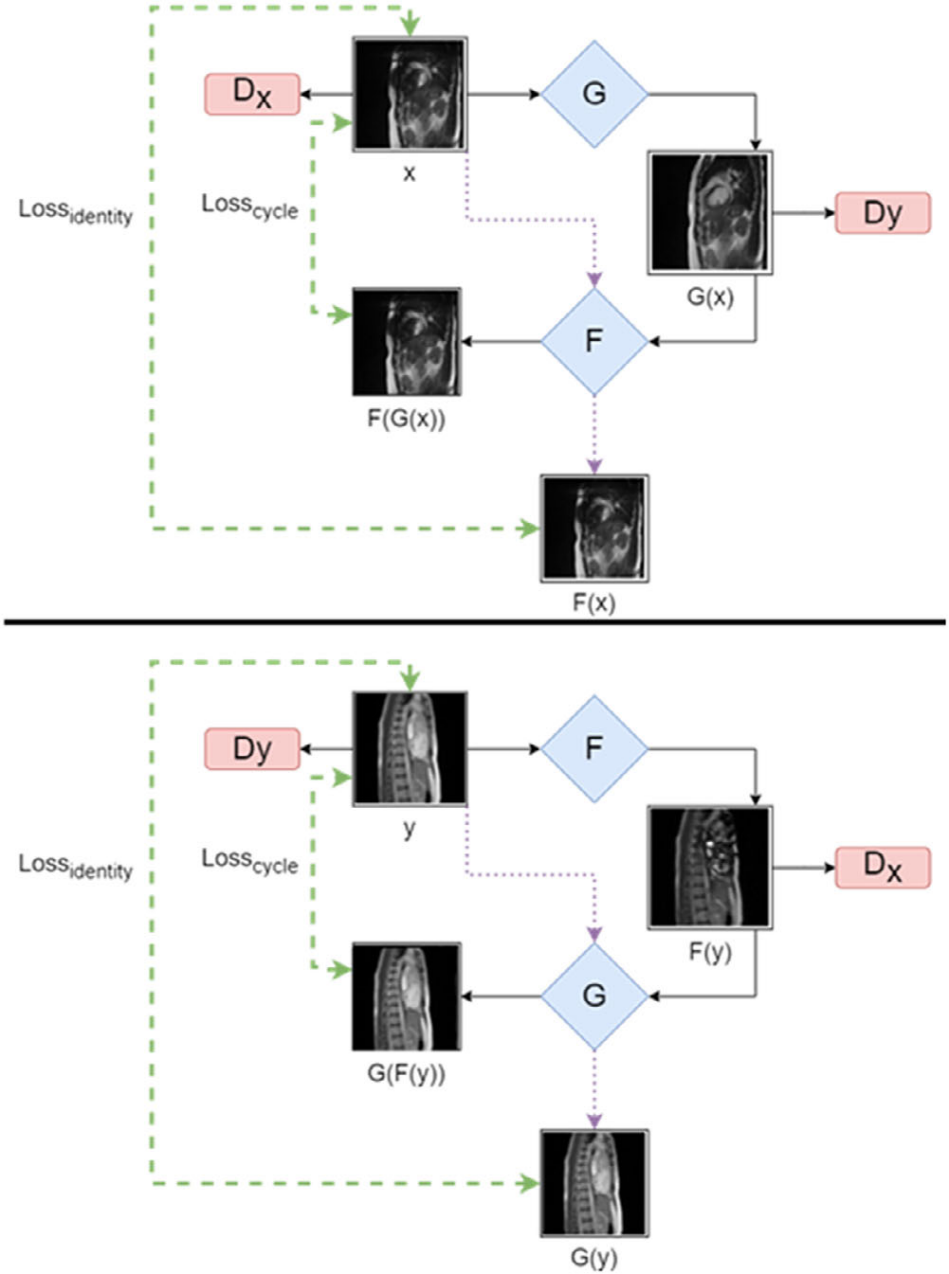
CycleGAN architecture shown as two flow charts. F and G are generator networks (shown in blue) where F maps from domain Y to domain X, and G maps from domain X to domain Y. D_x_ and D_y_ are discriminator networks (shown in red) that are trained to return a 0 for images not in the target domain (X for D_x_ and Y for D_y_) and return a 1 for images in the target domain. The identity and cycle loss inputs are indicated by a green dashed arrow. The identity loss image generation is shown via the purple dotted arrows. (Top) The pathway for an image, x, in domain X. X in this case corresponds to the domain containing ICD artifacts. (Bottom) The pathway for an image, y, in domain Y. Y corresponds to the image domain without artifacts.

The CycleGAN used two generators and two discriminators to translate images from one source domain to another without pairing images (Equations (1), (2), (3), (4), (5), (6)).^12^ Images from domain X were transformed to domain Y by training one generator to learn the mapping G: X → Y and the other to learn the mapping F: Y → X. The network architecture is shown in Figure 1 with the X domain corresponding to images with ICD artifacts and the Y domain corresponding to images without ICD artifacts.

The CycleGAN network was trained with a combination of three losses: adversarial loss; cycle‐consistency loss; and identity loss. The adversarial loss aims to produce images that are indistinguishable from the target domain and found to decrease the vanishing gradient problem compared to cross‐entry loss functions in GANs.^18^ The cycle consistency loss reduces the space of possible mapping functions and ensures the model retains consistency when mapping output images back to their initial domain. The identity loss preserves the content from the input domain.

The adversarial objective functions for generators G and F and discriminators D_y_ and D_x_ are:

(1) $$\min_{G}Loss_{G}\left(G\right)=E_{x∼p_{data}\left(x\right)}{\left[D_{y}\left(G\left(x\right)\right)-1\right]}^{2}$$

(2) $$\begin{array}{ccc} \min_{D_{y}}\;Loss_{D_{y}}\;\left(D_{y}\right) & = & E_{y∼p_{data}\left(y\right)}\;{\left[D_{y}\left(y\right)-1\right]}^{2}\\ & & +\;E_{x∼p_{data}\left(x\right)}\left[D_{y}{\left(G\left(x\right)\right)}^{2}\right] \end{array}$$

(3) $$\min_{F}\;Loss_{F}\;\left(F\right)=E_{y∼p_{data}\left(y\right)}\;{\left[D_{x}\left(F\left(y\right)\right)-1\right]}^{2}$$

(4) $$\begin{array}{ccc} \min_{D_{x}}\;Loss_{D_{x}}\;\left(D_{x}\right) & = & E_{x∼p_{data}\left(x\right)}\;{\left[D_{x}\left(x\right)-1\right]}^{2}\\ & & +\;E_{y∼p_{data}\left(y\right)}\left[D_{x}{\left(F\left(y\right)\right)}^{2}\right]\; \end{array}$$

where x ∈ X, y ∈ Y, and p_data_ is the data manifold to be learned. Minimizing these objective functions is equivalent to minimizing the Pearson χ^2^ divergence.^18^ These adversarial losses originated from the least squares GAN architecture and were shown to decrease the vanishing gradient problem compared to cross‐entry loss functions in GANs.^18^

The cycle consistency loss reduces the space of possible mapping functions and ensures the model retains consistency when mapping output images back to their initial domain. This loss can be broken down into a forward loss (F(G(x)) ≈ x) and a backward loss (G(F(y)) ≈ y). The full cycle consistency loss are:

(5) $$\begin{array}{ccc} Loss_{\text{cycle}}\left(G,F\right) & = & \lambda_{x}E_{x∼p_{\text{data}}\left(x\right)}\left[{∥F\left(G\left(x\right)\right)-x∥}_{1}\right]\\ & & +\;\lambda_{y}E_{y∼p_{\text{data}}\left(y\right)}{[∥G\left(F\left(y\right)\right)-y∥}_{1}] \end{array}$$

where λ_x_ and λ_y_ are weights that control the emphasis placed on either the forward or backwards loss.

An additional identity loss was added to the overall objective function to maintain G(y) ≈ y and F(x) ≈ x. This loss has the effect of making the model more conservative when encountering unknown content and discouraging the network from making large changes to inputs that are similar to images in the target domain. The identity loss was:

(6) $$\begin{array}{ccc} Loss_{identity}\left(G,F\right) & = & \lambda_{x}E_{x∼p_{data}\left(x\right)}\left[∥F\left(x\right)-x{∥}_{1}\right]\\ & & +\;\lambda_{identity}\left[\lambda_{y}E_{y∼p_{data}\left(y\right)}\left[∥G\left(y\right))-y{∥}_{1}\right]\right] \end{array}$$

where λ_identity_ scales the identity loss relative to the other loss functions.

### 2.2 Data acquisition

Fourteen healthy (Group A) volunteers (eight males), ages 22−74 (mean 42.4 ± 16.6 years) and weights 60−106 kg (mean 76.85 ± 11.61 kg) were recruited under Institutional Review Board approval to be imaged on a 0.35 T MRgRT system (ViewRay MRIdian, Oakwood Village, Ohio, USA). MRIs were performed using “MRI QA” mode with posterior and anterior torso receiver coils with gantry angle set to home (300°). Volunteers were positioned head‐first supine with the volunteer's arms at their sides to increase comfort.

Images were acquired using a 2D sagittal cartesian 4 frames/s (fps) bSSFP cine sequence (TR: 2.1 ms, TE: 0.91 ms, GRAPPA: 2, Partial Fourier: 5/8, Pixel Bandwidth: 1351 Hz/pixel, Matrix: 100 × 100, Field of View: 350 mm, 2 averages, 12‐bit depth). Volunteers were imaged with an MR Conditional ICD (Medtronic Visia AF MRI SureScan Model DVFB1D1, Minneapolis, Minnesota, USA) taped to their upper left pectoral region with leads (Models 6944−75 and Sprint Quattro DF‐1/IS‐1) running toward the ventricles to simulate an implanted ICD. Scans were repeated immediately after removing the ICD.

The images were aggregated into a domain with the ICD artifact and a domain without artifact. The images from Volunteers 13 and 14 were separated as testing data while the other twelve volunteer datasets were used for training. In addition, the data gathered from the 14 Group A volunteers was reused and resorted using a Leave‐One‐Out scheme, as obtaining new data was not feasible. We partitioned the data into a testing set from one volunteer and a training set from the other 13 volunteers (complement). The Leave‐One‐Out experiments were performed thrice with Group A Volunteers 3, 8, and 11 randomly selected as the test set for each run. Table 1 provides the number of training and test images for the different datasets.

**TABLE 1:** Dataset composition for Group A volunteers.

Volunteer	Training images		Testing images	
	No ICD	ICD	No ICD	ICD
3^a^	18,649	20,258	500	500
8^a^	18,649	20,258	500	500
11^a^	18,181	19,790	968	968
13^b^	18,149	19,758	1000	1000
14^b^	18,149	19,758	1000	1000

^a^ Trained with complement of volunteer data sets.

^b^ Trained with Volunteers 1−12 datasets.

### 2.3 Data analysis

CycleGAN training was performed using PyTorch and Python version 3.7 for 50 epochs (48 h). The weights for identity loss (λ_identity_), and forward and backward cycle‐consistency losses (λ_x_ and λ_y_) were empirically set to 0.7, 10, and 10, respectively. All images were upscaled to 256 × 256 pixels using bicubic interpolation. The two generators used a 9‐block ResNet architecture while the two discriminators used a 70 × 70 PatchGAN architecture.^19^

All image analysis was performed in MATLAB version 2022a. Image translation took 100 ms using an Intel i7‐9700K CPU with an NVDIA 3090 graphics card running Windows 11. For each ICD artifact image, the ground truth image (without ICD artifact) was created by calculating the multiscale structural similarity (MS‐SSIM) index for each possible pair of images and selecting the highest scoring pair.^20^

To assess target tracking for each test volunteer, a reference contour of the whole heart was drawn on the fifth image in the artifact‐free test dataset to ensure the bSSFP sequence had reached steady state. The first four images were still used in the analysis. Contours were then generated on each test image using an active contour algorithm.^21^ Dice similarity coefficients (DSC), target registration errors (TRE), and 95 percentile Hausdorff distances (95% HD) were calculated for both CycleGAN reconstructed images and ICD artifact images as surrogates for target tracking accuracy.

**FIGURE 2:**
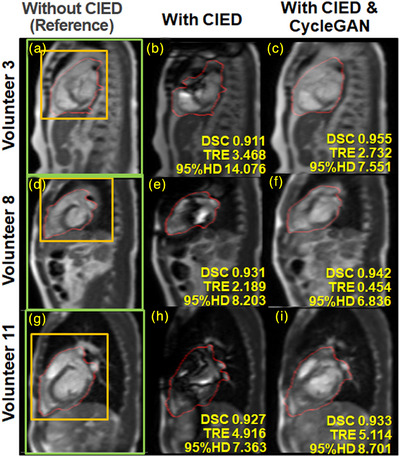
Images and tracking contours from Group A's Volunteer 3 (a‐c, 74‐year‐old female), Volunteer 8 (d‐f, 30‐year‐old male), and Volunteer 11 (g‐i, 58‐year‐old male). From left: MRIs acquired without the ICD present (a, d, and g), and with the ICD present (b, e, and h) and reconstructed using the CycleGAN (c, f, and i). ROIs for the test images are shown (in a, d, and g) for the heart contour generated from the active contouring algorithm (red); the heart and surrounding tissue (orange), and the whole‐body slice (green). Tracking metrics are shown in yellow for the heart contour at the bottom of the images acquired with the ICD in place. The CycleGAN generated images (c, f, and i) generally resulted in reduced susceptibility artifacts, higher DSC, and lower TRE and 95% HD values. The ICD locations are indicated by the labels b, c, e, f, h, and i.

**FIGURE 3:**
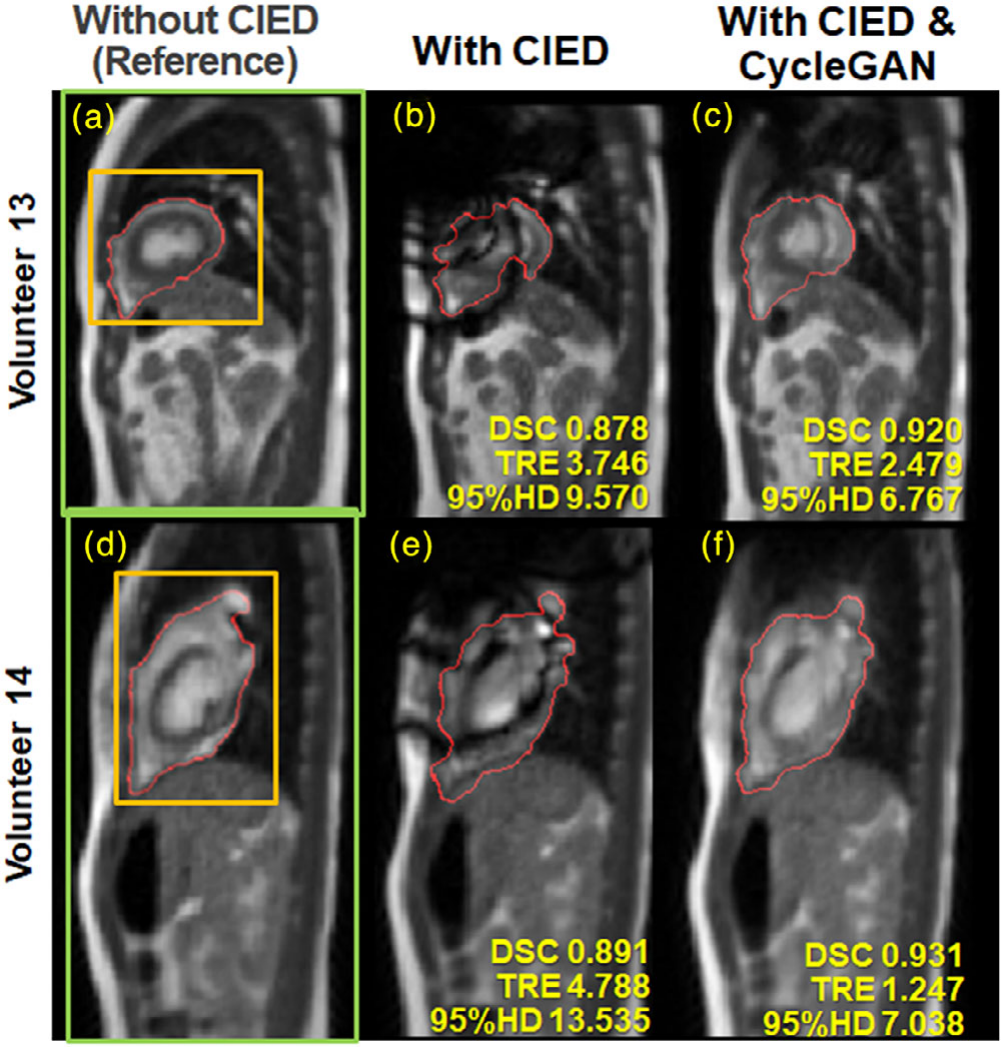
Images and tracking contours from Group A's Volunteer 13 (a‐c, 30‐year‐old male) and Volunteer 14 (d‐f, 31‐year old female). From left: MRIs acquired without the ICD present (a and d), and with the ICD present (b and e) and reconstructed using the CycleGAN (c, f). ROIs for the test images are shown (in a and d) for the heart contour generated from the active contouring algorithm (red); the heart and surrounding tissue (orange), and the whole‐body slice (green). Tracking metrics are shown in yellow for the heart contour at the bottom of the images acquired with the ICD in place. The CycleGAN generated images (c and f) generally resulted in reduced susceptibility artifacts, higher DSC, and lower TRE and 95% HD values. The ICD locations are indicated by the labels b, c, e, and f.

Image quality was scored by calculating the peak signal‐to‐noise ratios (PSNR), normalized root mean squared errors (nRMSE), and MS‐SSIM scores for the CycleGAN reconstructed images and the ICD artifact test images. Image quality metrics were calculated over three different regions of interest (ROIs): 1) the whole‐heart contour region from the paired reference image; 2) a bounding box enclosing the heart and surrounding tissue; and 3) a rectangular region that includes the whole body in the imaging slice to reduce the influence of background noise. The different ROIs are shown in Figures 2 and 3. Paired *t*‐tests were performed for all tracking and image quality metrics to assess significance.

The trained CycleGAN model was further tested on cine MRI datasets from three volunteers (Group B) to evaluate the robustness of the model. These datasets consisted of three additional 4 fps bSSFP cine MRI acquisitions from 2018. Because none of these cine MRIs were acquired with a ICD‐free reference set of images, the CycleGAN generated images were only compared qualitatively. The first dataset was acquired in a 61‐year‐old male VT patient with an implanted MR Conditional ICD (Medtronic Evera MRI Surescan Model DDMC3D4) and leads (Model 5076 CapSureFix Novus and Model 6935 Sprint Quattro Secure S) treated on the 0.35 T MRI‐Linac with arms up. The other two qualitative test cases were acquired with the same ICD and external placement used for Group A with arms down. The second cine MRI dataset was acquired from a 28‐year‐old male acquired on the 0.35 T MRI‐Linac. The third MRI dataset was acquired from a 38‐year‐old male volunteer scanned on a 0.35 T ViewRay MRI‐Cobalt‐60 radiotherapy system. The gantry angles were set to the home position (0° in 2018 for both systems).

## 3 RESULTS

Figures 2 and 3 show examples of the artifact reduction and tracking improvement using the trained CycleGAN model for test data sets from Group A. The susceptibility artifact from the ICD was substantially reduced for the CycleGAN reconstructed images. Image content not affected by the ICD was largely retained after using the CycleGAN.

Table 2 shows the tracking metric results for the whole‐heart contour ROIs, averaged over the images for each Group A volunteer used in testing. The mean DSC rose from 0.910 ± 0.018 to 0.935 ± 0.017 after CycleGAN‐reconstruction. The mean TRE dropped from 4.488 ± 1.647  to 2.877 ± 1.595 mm after CycleGAN‐reconstruction. The mean 95% HD dropped from 10.236 ± 2.605  to 7.700 ± 1.523 mm after CycleGAN‐reconstruction. The targets for TRE and 95% HD were 3.5 and 7 mm (1 and 2 pixels), respectively, based on a prior study.^22^

**TABLE 2:** Mean ± standard deviation of tracking metrics without (“ICD Artifact”) and with CycleGAN reconstruction for Group A Volunteers whole‐heart contours.

	DSC		TRE (mm)		95% HD (mm)	
Volunteer	ICD artifact	CycleGAN	ICD artifact	CycleGAN	ICD artifact	CycleGAN
3^a^	0.915 ± 0.010	0.953 ± 0.009	4.712 ± 1.278	3.243 ± 1.589	14.674 ± 1.892	7.326 ± 1.851
8^a^	0.923 ± 0.011	0.939 ± 0.011	2.459 ± 1.226	2.644 ± 1.755	9.801 ± 2.036	7.259 ± 1.671
11^a^	0.920 ± 0.007	0.939 ± 0.009	5.454 ± 0.780	4.037 ± 1.415	8.894 ± 1.033	8.044 ± 1.084
13^b^	0.886 ± 0.017	0.912 ± 0.016	3.893 ± 1.003	2.127 ± 0.881	9.034 ± 1.538	7.664 ± 1.862
14^b^	0.900 ± 0.013	0.929 ± 0.009	5.819 ± 1.649	2.103 ± 1.333	10.033 ± 1.808	7.883 ± 1.107
Mean	0.910 ± 0.018	0.935 ± 0.017	4.488 ± 1.647	2.877 ± 1.595	10.236 ± 2.605	7.700 ± 1.523

^a^ Trained with complement of volunteer data sets.

^b^ Trained with Volunteers 1−12 datasets.

Table 3 shows the mean image quality metrics for the whole‐body slice ROIs, averaged over the images for each Group A volunteer used in testing. The mean nRMSE dropped from 0.644 ± 0.178 to 0.420 ± 0.100 after CycleGAN‐reconstruction. The mean MS‐SSIM rose from 0.779 ± 0.074 to 0.819 ± 0.068 after CycleGAN‐reconstruction. The mean PSNR rose from 18.744 ± 1.562 to 22.368 ± 0.861 after CycleGAN‐reconstruction.

**TABLE 3:** Mean ± standard deviation of image quality metrics without (“ICD Artifact”) and with CycleGAN reconstruction for Group A Volunteers whole‐body slice ROIs.

	nRMSE		MS‐SSIM		PSNR	
Volunteer	ICD artifact	CycleGAN	ICD artifact	CycleGAN	ICD artifact	CycleGAN
3^a^	0.464 ± 0.092	0.277 ± 0.025	0.861 ± 0.026	0.897 ± 0.011	19.452 ± 1.544	23.786 ± 0.771
8^a^	0.622 ± 0.110	0.437 ± 0.025	0.779 ± 0.037	0.790 ± 0.012	18.555 ± 1.470	21.496 ± 0.426
11^a^	0.943 ± 0.095	0.511 ± 0.044	0.660 ± 0.036	0.717 ± 0.015	16.681 ± 0.764	21.987 ± 0.780
13^b^	0.585 ± 0.084	0.366 ± 0.022	0.786 ± 0.027	0.848 ± 0.011	18.140 ± 1.176	22.148 ± 0.485
14^b^	0.607 ± 0.032	0.509 ± 0.023	0.811 ± 0.009	0.843 ± 0.007	20.890 ± 0.452	22.421 ± 0.332
Mean	0.644 ± 0.178	0.420 ± 0.100	0.779 ± 0.074	0.819 ± 0.068	18.744 ± 1.562	22.368 ± 0.861

^a^ Trained with complement of volunteer data sets.

^b^ Trained with Volunteers 1−12 datasets.

The image quality metrics for the heart contour and heart and surrounding tissue ROIs are provided in the Supplemental Materials. Histograms for the tracking metrics for the heart contour ROI and image quality metrics for the whole‐body slice ROI are also presented in the Supplemental Materials.

**FIGURE 4:**
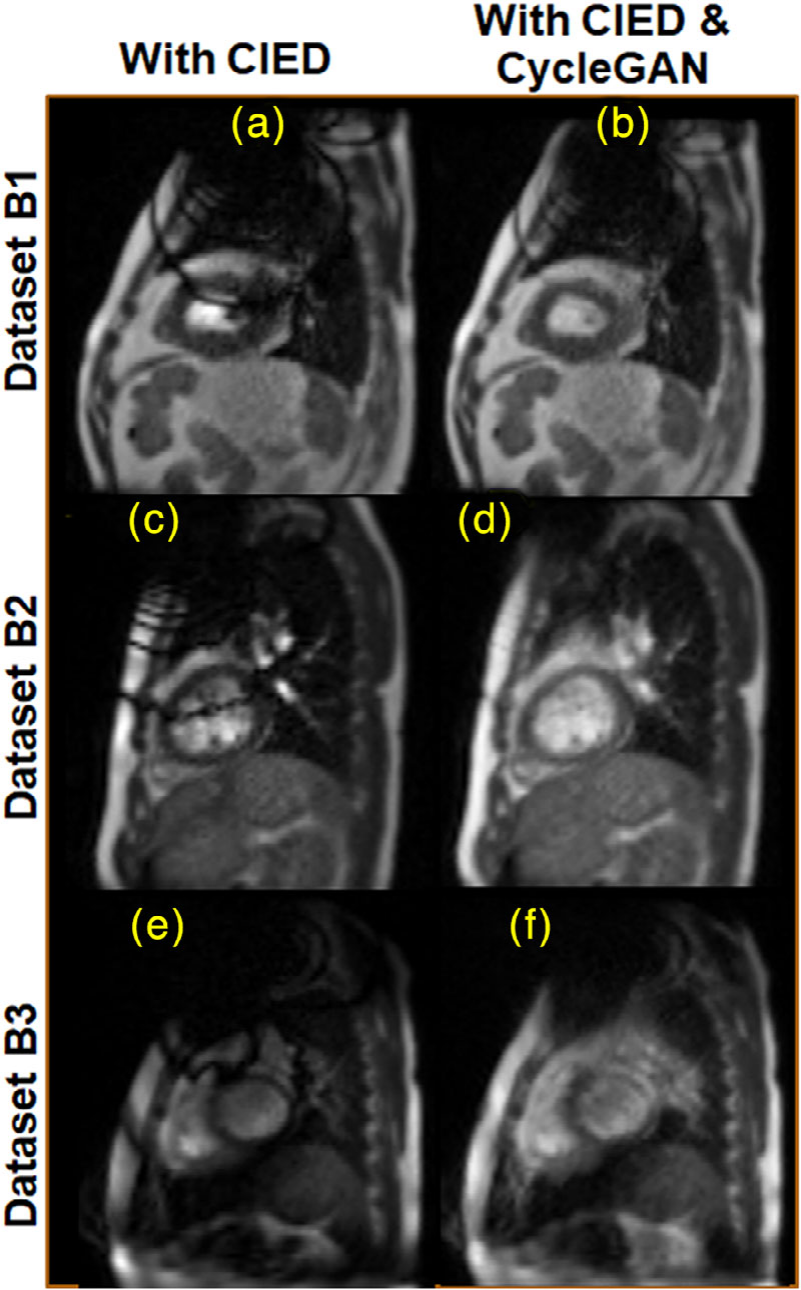
Application of CycleGAN to the three Group B ICD datasets not used in model training or testing. Sagittal 4 fps cine bSSFP MRIs are shown without (a, c, e) and with (b, d, f) CycleGAN processing. (a, b) 61‐year‐old male VT patient with implanted ICD. (c, d) 28‐year‐old and (e, f) 38‐year‐old healthy male volunteers. The latter (e, f) was scanned on a 0.35 T ViewRay MRI‐Cobalt‐60 MRgRT. The position of the ICD is indicated by the letter labels (a‐f).

All paired *t*‐tests had *p* < < 0.001 indicating a significant benefit in the CycleGAN generated images compared with the ICD artifact images. CycleGAN artifact‐reduced images from the three Group B cine MRI datasets without an ICD reference are shown in Figure 4. All three show a reduction in banding artifacts near the heart.

## 4 DISCUSSION

The objective of this work was to investigate using CycleGAN to reduce ICD susceptibility artifacts in bSSFP cines commonly used in MRgRT. The CycleGAN reconstruction restored visible anatomy around the tracking target (heart) that was lost to the artifact. The CycleGAN‐reconstructed images exhibited higher DSC scores and reduced TRE and HD values, implying improved tracking performance. The CycleGAN also improved image quality with the ICD present as indicated by the reduction in nRMSE and increases in both PSNR and MS‐SSIM.

The main limitation of this study is the small sample size (i.e., number of data sets). Additional training data should further improve the CycleGAN artifact reduction capabilities. Since the training data came from either 12 volunteers (Volunteers 13 and 14 test cases) or 13 volunteers (Volunteers 3, 8, and 11 test cases), the training data may have a high degree of correlation between training images.

In addition, we used a single model and placement of ICD for training. ICDs use an iron core step‐up transformer to generate the high voltages for administering defibrillator shocks. The transformer is ferromagnetic and is responsible for most of the artifact created by the ICD. The transformer is located next to the lead connector block regardless of the make or model. Therefore, the variation in the position of the transformer within the ICD will be smaller than the variation in the ICD's left subcutaneous pectoral position.

An additional limitation of this study is that the test image pairs used to evaluate performance were imperfect. Initially, we trained the supervised conditional GAN pix2pix with our image pairs but the test results were unsatisfactory particularly within the abdominal cavity. Therefore, we pursued the unsupervised CycleGAN and found it was better suited for imperfect pairs. The test image pairing based on MS‐SSIM scores resulted in data pairs that visually tracked the cardiac and respiratory cycle well. However, small differences in the lung vasculature and abdominal structures remained in place due to slight differences in image positioning, coil placement, and physiological motion. While great care was taken to keep the volunteers and receiver coils from moving when removing the ICD, deviations between the data acquired with the ICD present and absent are expected. Generating high quality, realistic, synthetic susceptibility artifacts on digital, anatomically correct MRI phantoms could provide a more robust data source.

We trained the CycleGAN with arms down. However, cardiac patients are treated with both arms raised that causes the ICD to shift toward the head. Ideally, the model should be trained with the arm position relevant to the treatment plan. Nevertheless, the image artifacts from the volunteers with the externally positioned ICD (Figures 2 and 3) resemble artifacts from the patient with the implanted ICD (Figure 4 Dataset B1).

Another limitation was the data were acquired at a single gantry angle without B_0_ shimming. Phantom measurements indicated that for our MRI‐Linac, the effect of the gantry angle on MS‐SSIM was small (standard deviation < 5 × 10^−5^). However, B_0_ shimming at each gantry angle can reduce the ICD artifacts distal to the ICD.

The CycleGAN method can be applied to other cine sequences used in MRgRT (e.g., ViewRay's 8 fps radial cine sequence) if trained with the respective sequence and running on a fast GPU processor. Real‐time reconstruction times of < 30 ms are anticipated after hardware and software optimization (e.g., delay up‐sampling, and reduce PatchGAN and ResNet blocks).^23^ However, we use the 4 fps cartesian cine sequence by default for patients with metal implants since its specific absorption rate (SAR) is roughly half of the 8 fps sequence and treatments may require over an hour of continuous MRI acquisition.

The implementation of GANs directly into the Siemens ICE reconstruction pipeline is challenging due to the lack of support for programming languages regularly used for machine learning (e.g., Python, MATLAB, and R). Integration of the CycleGAN with the ViewRay architecture should be straightforward using the treatment delivery computing unit (TDCU). The TDCU is a multiple GPU based processor that currently post‐processes images and performs tracking. Open‐source reconstruction frameworks with Python 3 support, such as Gadgetron, are an alternative for researchers.^24^

Next steps include applying the method to 1.5 T where susceptibility artifacts will be more severe than 0.35 T. In addition, alternative supervised learning networks may be substituted for the unsupervised CycleGAN used herein.

## AUTHOR CONTRIBUTIONS

A. Curcuru acquired the data, developed the methods, performed the data analysis, and wrote the draft manuscript. D. Yang funded the study, developed the methods, and edited the manuscript. H. An contributed to the methods and edited the manuscript. P.S. Cuculich and C.G. Robinson developed the purpose of the study, provided the implantable defibrillator and patient volunteer, and edited the manuscript. H.M. Gach funded the study, recruited the volunteers, developed the methods, and revised the manuscript.

## CONFLICT OF INTEREST STATEMENT

The authors declare no conflicts of interest.

## Supporting information

Supporting Information
