# Supplementary material for: Technical note: Minimizing CIED artifacts on a 0.35 T MRI‐Linac using deep learning
Source: J Appl Clin Med Phys. 2024 Feb 18;25(3):e14304. doi: 10.1002/acm2.14304 (PMC10929994; doi:10.1002/acm2.14304)
Supplement: Supplementary file 1 — Supporting Information [file ACM2-25-e14304-s001.pdf]

SUPPLEMENTAL MATERIALS

RESULTS

Image quality (normalized root mean square error (nRMSE), peak signal-to-noise ratio (PSNR), and multiscale structural similarity (MS-SSIM)) scores for the five volunteers and the whole-heart contour and the heart and surrounding tissue regions of interest (ROIs).

TABLE S1 Mean ± standard deviation nRMSE without ("ICD Artifact") and with CycleGAN reconstruction for Group A Volunteers.

Volunteer	Whole-Heart Contour ROI		Heart and Surrounding Tissue ROI	
	**ICD Artifact**	**CycleGAN**	**ICD Artifact**	**CycleGAN**
**3***	1.390 ± 0.169	0.550 ± 0.099	1.197 ± 0.157	0.277 ± 0.025
**8***	2.016 ± 0.240	1.042 ± 0.105	1.614 ± 0.176	0.437 ± 0.025
**11***	2.147 ± 0.154	1.037 ± 0.165	2.689 ± 0.199	0.511 ± 0.044
**13****	2.573 ± 0.297	1.281 ± 0.155	1.469 ± 0.151	0.366 ± 0.022
**14****	1.950 ± 0.112	1.136 ± 0.122	1.471± 0.075	0.509 ± 0.023
**Mean**	2.015 ± 0.425	1.009 ± 0.275	1.688 ± 0.580	0.420 ± 0.100

* Trained with complement of volunteer data sets. ** Trained with Volunteers 1-12 datasets.

TABLE S2 Mean ± standard deviation MS-SSIM without ("ICD Artifact") and with CycleGAN reconstruction for Group A Volunteers.

Volunteer	Whole-Heart Contour ROI		Heart and Surrounding Tissue ROI	
	**ICD Artifact**	**CycleGAN**	**ICD Artifact**	**CycleGAN**
**3***	0.948 ± 0.010	0.983 ± 0.004	0.935 ± 0.013	0.897 ± 0.011
**8***	0.931 ± 0.016	0.974 ± 0.004	0.881 ± 0.019	0.790 ± 0.012
**11***	0.863 ± 0.024	0.950 ± 0.011	0.831 ± 0.025	0.717 ± 0.015
**13****	0.958 ± 0.008	0.987 ± 0.002	0.893 ± 0.014	0.848 ± 0.011
**14****	0.947 ± 0.008	0.980 ± 0.004	0.908 ± 0.005	0.843 ± 0.007
**Mean**	0.929 ± 0.038	0.975 ± 0.015	0.890 ± 0.038	0.819 ± 0.068

* Trained with complement of volunteer data sets. ** Trained with Volunteers 1-12 datasets.

TABLE S3 Mean ± standard deviation PSNR without ("ICD Artifact") and with CycleGAN reconstruction for Group A Volunteers.

Volunteer	Whole-Heart Contour ROI		Heart and Surrounding Tissue ROI	
	**ICD Artifact**	**CycleGAN**	**ICD Artifact**	**CycleGAN**
**3***	24.389 ± 0.922	32.245 ± 1.609	23.333 ± 0.988	23.786 ± 0.771
**8***	23.872 ± 1.020	29.586 ± 0.962	22.311 ± 0.935	21.496 ± 0.426
**11***	18.796 ± 0.578	25.203 ± 1.348	20.057 ± 0.560	21.987 ± 0.780
**13****	26.875 ± 1.262	32.927 ± 0.971	24.837 ± 1.052	22.148 ± 0.485
**14****	25.403 ± 0.549	30.137 ± 0.907	23.607 ± 0.417	22.421 ± 0.332
**Mean**	23.867 ± 3.058	30.020 ± 3.033	22.829 ± 1.792	22.368 ± 0.861

* Trained with complement of volunteer data sets. ** Trained with Volunteers 1-12 datasets.

Histograms

The histograms for the tracking (Dice similarity coefficients (DSC), target registration error (TRE), and 95 percentile Hausdorff distance (95% HD) and image quality (normalized root mean square error (nRMSE), peak signal-to-noise ratio (PSNR), and multiscale structural similarity (MS-SSIM)) metrics. Figure S1 presents the histograms for test datasets from Left-One-Out Volunteers 3, 8, and 11. Figure S2 presents the histograms for test datasets from Volunteers 13 and 14.

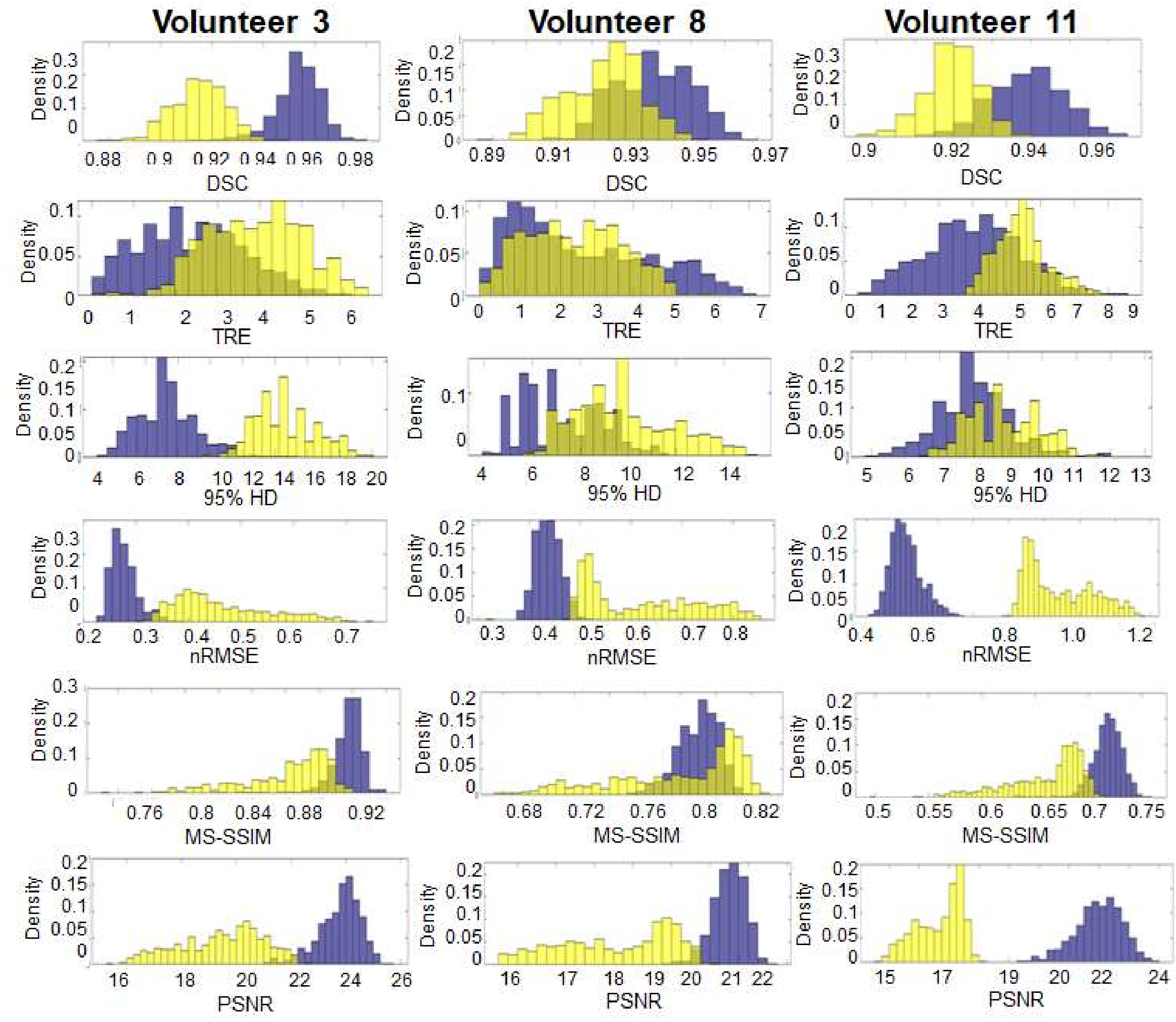

FIGURE S1 Tracking and image quality metric histograms for the ICD artifact images (yellow) and images using CycleGAN artifact reduction (purple) for Volunteers 3, 8, and 11 datasets used for testing. Tracking metrics were calculated over the whole-heart contour. Image quality metrics were calculated over the whole-body slice ROI.

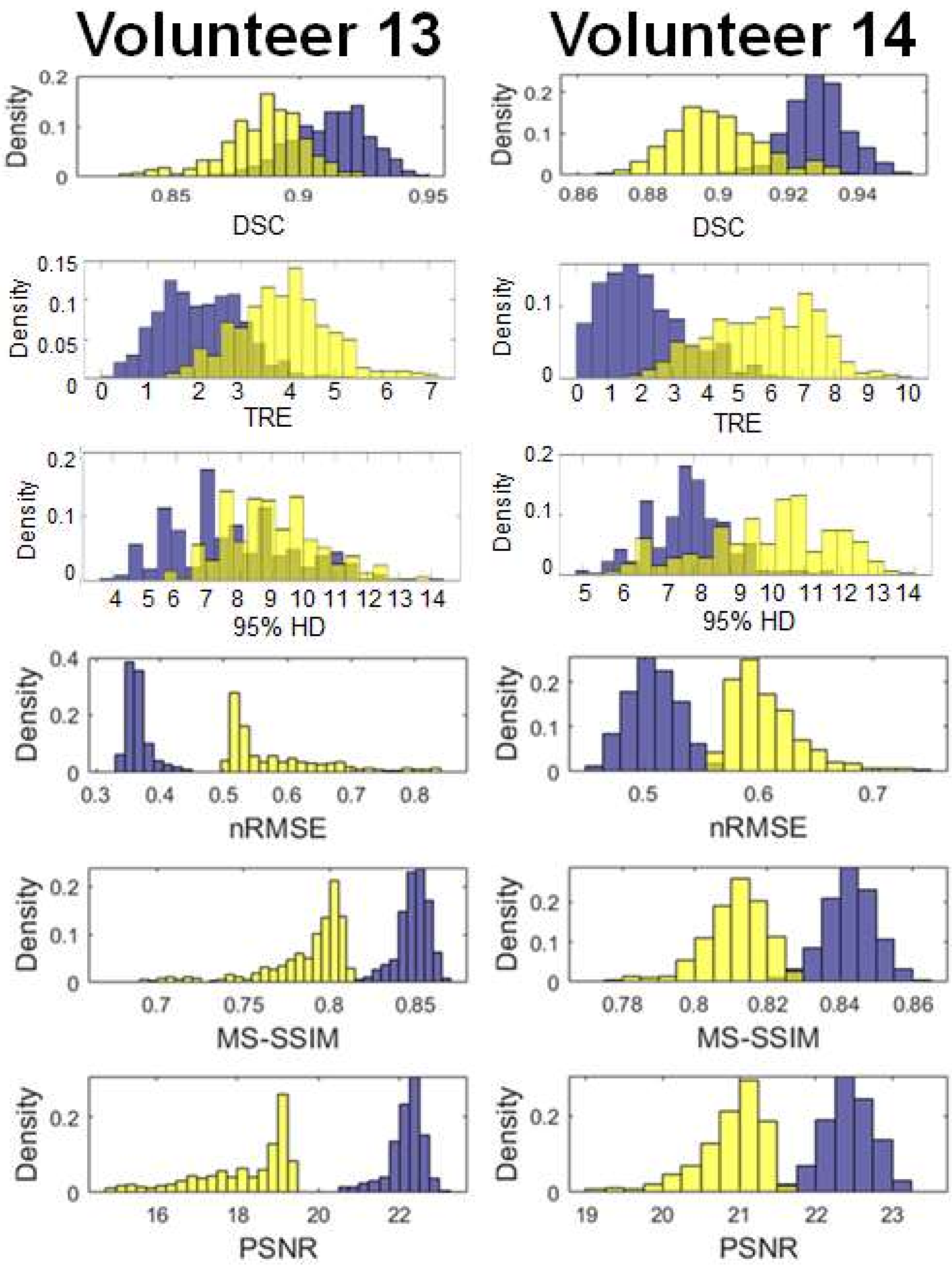

FIGURE S2 Tracking and image quality metric histograms for the ICD artifact images (yellow) and images using CycleGAN artifact reduction (purple) for Volunteers 13 and 14 datasets used for testing. Tracking metrics were calculated over the whole-heart contour. Image quality metrics were calculated over the whole-body slice ROI.
